# Sonic Hedgehog/Gli1 Signaling Pathway Regulates Cell Migration and Invasion via Induction of Epithelial-to-mesenchymal Transition in Gastric Cancer

**DOI:** 10.7150/jca.42900

**Published:** 2020-04-06

**Authors:** Bin Ke, Xiao-Na Wang, Ning Liu, Bin Li, Xue-Jun Wang, Ru-Peng Zhang, Han Liang

**Affiliations:** Department of Gastric Cancer, Tianjin Medical University Cancer Institute and Hospital, National Clinical Research Center for Cancer, Tianjin's Clinical Research Center for Cancer, Key Laboratory of Cancer Prevention and Therapy of Tianjin, Tianjin, 300060, P.R. China

**Keywords:** sonic hedgehog, epithelial-mesenchymal transition, gastric cancer, invasion, migration

## Abstract

**Background**: The aberrant activation of the Sonic hedgehog (Shh) signaling pathway is involved in progression of several types of cancer, including gastric cancer (GC). However, it remains uncertain whether it also plays a critical role in promoting cancer initiation and progression by inducing epithelial-to-mesenchymal transition (EMT) in GC. Thus, the aim of the present study was to determine whether the Shh pathway is involved in GC, and to investigate the function of the Shh pathway in the induction of EMT in GC.

**Materials and methods**: The expression levels of Shh pathway members and EMT markers were examined in GC tissues by immunohistochemistry. The association between these factors and patient clinicopathological characteristics was analyzed. In addition, Gli-antagonist 61 (GANT61) was used to block Shh/Gli1 pathway activity, and recombinant Shh proteins (N-Shh) were used to activate the Shh pathway in GC cells. Wound healing and Transwell invasion and migration assays were performed to assess the effects of the Shh pathway on the migration and invasion of GC cells *in vitro*. Furthermore, western blot analysis was used to examine the changes in protein expression.

**Results**: The results demonstrated that these Shh/Gli1 pathway members were upregulated in GC tissues, and that Gli1 upregulation was associated with tumor progression and a poor prognosis. Gli1 expression was negatively associated with E-cadherin (E-Cad) expression, and positively with Vimentin (VIM) expression in GC specimens. Further analysis revealed that when the Shh/Gli1 pathway was activated, the migratory and invasive abilities of GC cells were enhanced, and the expression levels of Gli1 and VIM were increased, while E-Cad expression was decreased. Opposite results were observed when the Shh/Gli1 pathway was blocked by GANT61.

**Conclusions**: The present study indicated that the Shh/Gli1 pathway exhibits an abnormal activation pattern in GC with possible predictive and prognostic significance. The Shh/Gli1 pathway may promote the migratory and invasive potential of GC cells by inducing EMT. The Shh/Gli1 pathway can thus be considered as a potential therapeutic target for GC.

## Introduction

Gastric cancer (GC) is the fifth most frequent type of cancer and the third primary cause of cancer-associated mortality worldwide with approximately 783 thousand deaths reported annually 1. Despite recent improvements in diagnosis and therapy, the prognosis of patients with advanced-stage GC remains dismal, with the 5-year overall survival rate being <30% 2, 3. Local tumor invasion and distant metastasis may be the major reasons for the poor survival of patients with advanced GC 4. Therefore, understanding the exact molecular mechanisms underlying the development and progression of GC is important for the development of novel treatment targets for patients with GC.

The hedgehog (Hh) signaling pathway is a well-conserved pathway across various species that plays a critical role during embryonic development and tissue morphogenesis in adults 5, 6. In humans, the Hh pathway is composed of Hh ligands, two transmembrane protein receptors [Patched receptor (Path) and Smoothened receptor (Smo)], and the five-zinc finger transcription factor Glis 7. In the canonical Hh pathway, when the Hh ligand is absent, Ptch suppresses the activity of Smo, and thus inhibits the cleavage of Gli, blocking Hh pathway activation. When the Hh ligand is present, it binds to Ptch, relieving the inhibition of Smo and resulting in the activation of Gli transcription factor. Activated Gli translocates to the nucleus and subsequently regulates the transcription of Gli target genes 8. In mammals, three ligands have been identified in the Hh pathway: Sonic hedgehog (Shh), Desert hedgehog (Dhh) and Indian hedgehog (Ihh) 7. Among these three ligands, Shh is the most thoroughly studied one, as well as the most important one 9, 10. Recently, the ectopic activation of the Shh pathway has been detected in a number of human malignancies 11, 12. Numerous reports have indicated that the Shh pathway plays a crucial role in GC initiation, progression and metastasis, and it has also been shown to be associated with patient prognosis 13-15. Therefore, a deeper understanding of this pathway in tumor invasion and metastasis may provide great promise for GC therapy.

Epithelial-to-mesenchymal transition (EMT) exerts marked effects on cellular transdifferentiation during embryonic development 16, and has been characterized as cells acquiring mesenchymal spindle-like morphology and the loosening of intercellular cohesion 17, 18. The characteristic features of EMT are the decreased expression of the cell adhesion molecule, E-cadherin (E-Cad), and the enhanced expression of the mesenchymal marker, Vimentin (VIM) 19, 20. Previous studies have demonstrated that EMT is a vital process in tumor progression and metastasis, and is associated with a poor prognosis and tumor aggressiveness in several human epithelial cancers, including GC 21-24. The identification of factors that induce EMT would greatly contribute to understanding the mechanisms of invasion and metastasis in GC, and may aid the development of effective approaches for the prevention and therapy of this malignancy.

Recently, several studies have demonstrated that the abnormal activation of the Shh pathway affects EMT in several types of cancers, such as hepatocellular carcinoma and lung cancer 25, 26. However, the association between the Shh pathway and EMT in GC has rarely been reported, and whether or not the Shh pathway may promote cancer development and progression by inducing EMT in GC has not yet been investigated, at least to the best of our knowledge. In the present study, we analyzed the expression of Shh pathway components and patient clinicopathological features and prognosis in GC tissues to evaluate whether the Shh pathway is involved in GC. In addition, the activation of the Shh pathway was regulated by treatment with exogenous recombinant Shh protein (N-Shh) or Gli-antagonist 61 (GANT61), a Gli specific inhibitor, to further explore the detailed mechanisms of the role of the Shh pathway in the regulation of EMT in GC. This study may indicate the potential clinical significance of the Shh pathway and EMT in GC.

## Materials and methods

### Patients and specimens

A total of 178 GC specimens and 89 adjacent non-tumor tissues were collected from patients diagnosed with GC at TianJin Medical University Cancer Institute and Hospital (TianJin, China) between March 2010 and April 2013. All the patients underwent radical gastrectomy with D_2_ lymphadenectomy. None of the patients had undergone chemotherapy, radiotherapy, or any other treatment for cancer prior to surgery. The surgical tissues were formalin-fixed and paraffin-embedded. The clinicopathological data were collected and analyzed. The pathological tumor staging was classified according to the 8th edition of the American Joint Committee on Cancer TNM staging system for GC 27.

This study was approved by the Ethics Committee of TianJin Medical University Cancer Institute and Hospital (Tianjin, China).

### Cell culture and treatment

The human gastric adenocarcinoma cell lines, KATO, MKN-45, SGC-7901, MGC-803, AGS, N87 and BGC-823, and the human gastric epithelial cell line, GES-1, were obtained from the American Type Culture Collection (ATCC). The cell lines were maintained in RPMI-1640 (Life Technologies; Thermo Fisher Scientific, Inc.) medium with 10% fetal bovine serum (FBS) (Thermo Fisher Scientific, Inc.), and 1% penicillin-streptomycin solution (Thermo Fisher Scientific, Inc.) at 37˚C in a humidified atmosphere of 5% CO_2_.

All cultures were routinely grown to ~70% confluence one day before treatment. For the inhibition of the Shh pathway, the cultured cells were incubated with GANT61 (Selleck Chemicals) at concentrations of 20 μM for 48 h. N-Shh (250 μg/ml) (Abcam) was used to activate this pathway, with the vehicle (DMSO) as controls.

### Immunohistochemistry

Immunohistochemistry were performed as previously described 28. In brief, 4-μm-thick paraffin-embedded specimens were deparaffinized in xylene and rehydrated through graded methanol. Endogenous peroxidase was blocked with 3% hydrogen peroxide for 30 min at room temperature. The sections were then incubated with primary antibodies overnight at 4˚C. After rinsing, the slides were incubated with the horseradish peroxidase- linked secondary antibody for 1 h at room temperature and visualized using 3,3'-diaminobenzidine and counterstained with hematoxylin. The primary antibodies were as follows: anti-Gli1 antibody (cat. no. ab49314, 1:200 dilution, Abcam), anti-SMO antibody (cat. no. ab113438, 1:200 dilution, Abcam), anti-PTCH1 antibody (cat. no. ab53715, 1:200 dilution, Abcam), anti-SHH antibody (cat. no. ab53281, 1:100 dilution, Abcam), anti-E-Cad antibody (cat. no. ab40772, 1:200 dilution, Abcam) and anti-VIM antibody (cat. no. ab92547, 1:200 dilution, Abcam). Negative controls were used by replacing the primary antibody with PBS. The staining result was evaluated by integrating the intensity of positive staining (0, negative; 1, weak; 2, moderate; 3, strong) and the percentage of positive stained cells (0, 0%; 1, 1-25%; 2, 26-50%; 3, 51-75%; 4, 76-100%). The final scores were calculated by adding the score of the staining intensity and the percentage of positive cells. A total score of ≤3 was noted as negative expression and a score of >3 were designated as positive expression.

### Western blot analysis

Total protein was extracted from the KATO, MKN-45, SGC-7901, MGC-803, AGS, N87, BGC-823 and GES-1 cells using the mammalian protein extraction reagent, RIPA lysis buffer (Beyotime Biotechnology). The protein concentration was determined using the BCA kit method (Pierce; Thermo Fisher Scientific, Inc.).

Approximately 40 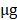
proteins were loaded and separated by 10% sodium dodecyl sulphate-polyacrylamide electrophoresis (SDS-PAGE) gel and transferred onto polyvinylidene difluoride membranes (EMD Millipore). The membranes were blocked with 5% skimmed milk for 1 h at room temperature and then incubated with primary antibodies at 4˚C overnight. After washing, the membranes were exposed to horseradish peroxidase (HRP)-labeled secondary antibodies (Zhongshan Jinqiao Biotechnology) for 2 h at room temperature. The protein bands were detected with the ECL (Thermo Fisher Scientific, Inc.) method, and the bands were quantified by densitometry using Quantity One software (Bio-Rad) using GAPDH as a loading control.

The antibodies against Gli1 (cat. no. ab49314), E-Cad (cat. no. ab40772), VIM (cat. no. ab92547) were obtained from Abcam, and the GAPDH antibody was obtained from Zhongshan Jinqiao Biotechnology.

### Wound healing assay

A total of 3x10^5^ cells were planted into a six-well plate and incubated for 24 h to ensure the cells grew to 95-100% confluence, and monolayer cells were then scratched using a 200 µl pipette tip. The wounded cells were washed with PBS three times and cultured in serum-free RPMI-1640 medium for 48 h. An inverted microscope was used to measure the average distance between the two wound edges. Duplicate wells were examined for each condition, and each experiment was performed three times.

### Cell migration and invasion assays

The migratory and invasive potential of the cells *in vitro* were determined using a Transwell chamber (8-µm pore size for 24-well plate) (Corning, Inc.) assay, with or without Matrigel coating (BD Biosciences). For the migration assay, a total of 1x10^5^ cells/well were suspended in serum-free RPMI-1640 and plated in the upper Transwell chambers, and 500 µl RPMI-1640 medium with 10% FBS were then added to the lower chamber as a chemoattractant. For the invasion assay, the upper side of the Transwell membrane was coated with diluted Matrigel first and cultured with 2x10^5^ cells/well. Subsequent to incubation at 37°C for 24 h, the cells that traversed to the lower side of membrane were fixed with 100% methanol for 20 min and stained with 0.5% crystal violet. The numbers of cells were counted in five random fields under an inverted microscope and the mean number was calculated. Each experiment was performed three times.

### Statistical analysis

All statistical analyses were conducted using SPSS statistical software version 22.0 (IBM Corp.). The Chi-square test was applied for all categorical variables, and the Student's t-test was used to compare continuous variables between two groups. The associations between the variables were assessed by calculating the odds ratio (OD) with the 95% confidence interval (CI). Kaplan-Meier analysis was used for survival analysis, and the log-rank test was used to determine significance. A multivariate survival analysis was performed for all parameters that were significant in the univariate analyses using the Cox regression model. A P-value <0.05 was considered to indicate a statistically significant difference.

## Results

### Shh pathway is aberrantly activated in GC

**Figure 1:**
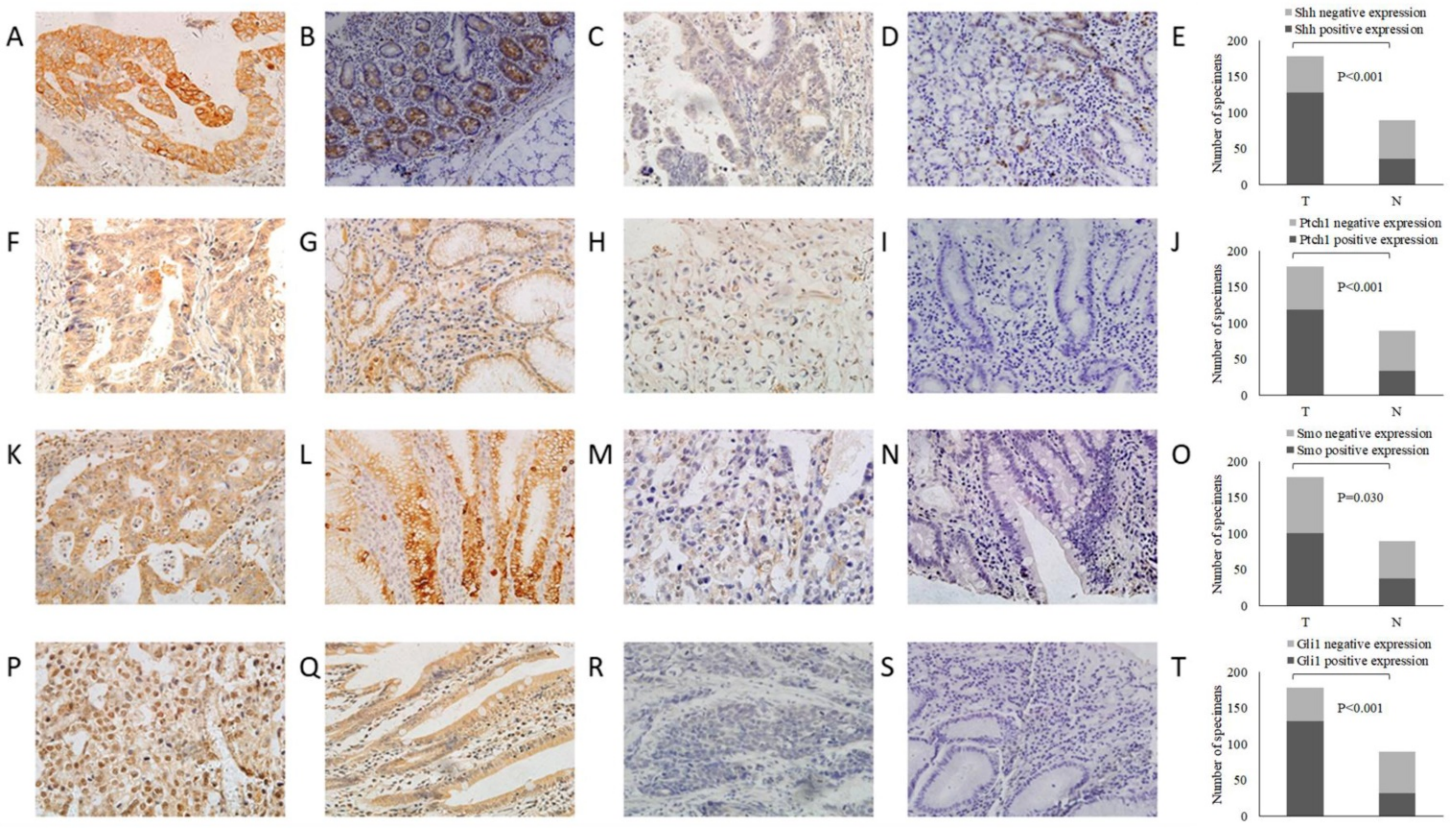
Representative images of Shh, Ptch1, Smo and Gli1 expression by immunohistochemistry (magnification, x400). (A) Shh positive expression in GC tissues, (B) Shh positive expression in adjacent non-tumor tissues, (C) Shh negative expression in GC tissues, (D) Shh negative expression in adjacent non-tumor tissues, (E) comparisons of Shh expression in GC tissues and adjacent non-tumor tissues, (F) Ptch1 positive expression in GC tissues, (G) Ptch1 positive expression in adjacent non-tumor tissues, (H) Ptch1 negative expression in GC tissues, (I) Ptch1 negative expression in adjacent non-tumor tissues, (J) comparisons of Ptch1 expression in GC tissues and adjacent non-tumor tissues, (K) Smo positive expression in GC tissues, (L) Smo positive expression in adjacent non-tumor tissues, (M) Smo negative expression in GC tissues, (N) Smo negative expression in adjacent non-tumor tissues, (O) comparisons of Smo expression in GC tissues and adjacent non-tumor tissues, (P) Gli1 positive expression in GC tissues, (Q) Gli1 positive expression in adjacent non-tumor tissues, (R) Gli1 negative expression in GC tissues, (S) Gli1 negative expression adjacent non-tumor tissues, (T) comparisons of Gli1 expression in GC tissues and adjacent non-tumor tissues. T, GC tissue; N, adjacent non-tumor tissue; GC, gastric cancer.

In order to assess Shh pathway activation in GC, we first used immunohistochemistry to examine the protein expression of Shh pathway members (Shh, Ptch, Smo and Gli1) in GC and adjacent non-tumor tissue samples (Fig. 1). The Shh and Ptch1 proteins were positively expressed in the cytoplasm, and it was found that 71.9% (128/178) and 66.9% (119/178) of the GC tumor specimens stained positively, which were significantly higher in the GC tissues compared with the adjacent non-tumor tissues (71.9 vs. 43.8%; 66.9 vs. 38.2%, P<0.001, respectively). Smo expression was located mainly in the cytoplasm or on the cell membrane. In GC tissues, 56.7 % (101/178) of specimens were positive for Smo staining, which was significantly higher than that observed in adjacent non-tumor tissue specimens (42.7 %; 38/89, P=0.030). Gli1-positive expression was observed mainly in the nucleus or cytoplasm. The results revealed that 74.2 % (132/178) of the GC tissues were positively stained for Gli1, which was a much higher percentage than that detected in the adjacent non-tumor tissues (36.0%; 32/89; P<0.001). These findings indicated that the expression of these Shh pathway members was markedly upregulated in GC tissues compared with adjacent non-tumor tissues (Table 1).

Subsequently, the association between the expression of Shh, Ptch1, Smo and Gli1 in GC tissues was evaluated. The results are shown in Table 2 and it was indicated that there were significant positive associations between every two factors (all OR>1, P<0.05).

### Aberrant activation of the Shh pathway is associated with adverse clinicopathological factors and a poor clinical outcome in patients with GC

In order to estimate the functions of the Shh/Gli1 pathway in the progression of GC, the association between the expression of these Shh pathway members and the clinicopathological features of GC was analyzed. As shown in Table 3, no significant associations were found between the Shh, Ptch1, Smo and Gli1 expression levels and age, sex, or tumor size (P>0.05). The overexpression of Shh, Ptch1 and Gli1 was significantly associated with a low histological grade and a deeper invasion depth of GC (P<0.05). Furthermore, the data demonstrated that Shh, Ptch1, Smo and Gli1 overexpression was markedly associated with a higher incidence of Lymph node metastasis and a more advanced TNM stage in GC (P<0.05). Taken together, these results indicated that patients with GC with Shh pathway overactivation may have a deeper tumor invasion, more frequent lymph node metastasis and an advanced TNM stage. The overactivation of the Shh pathway is associated with an aggressive malignant phenotype.

**Figure 2:**
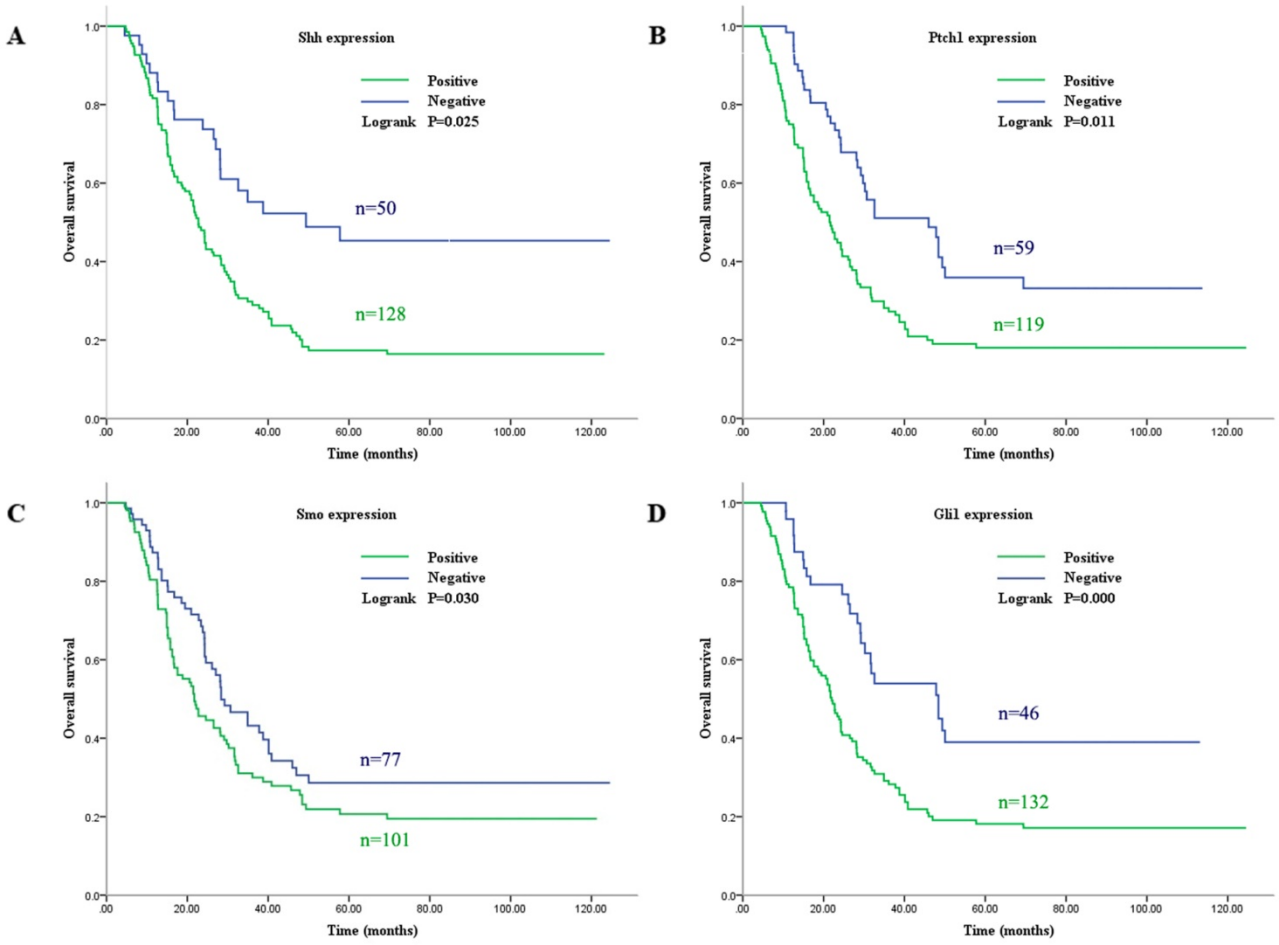
Survival curve for the OS of patients with GC according to Shh, Ptch1, Smo and Gli1 expression. (A) Shh, (B) Ptch, (C) Smo and (D) Gli1. OS, overall survival; GC, gastric cancer.

Subsequently, the association between the expression level of these Shh pathway components and the patient survival rate was determined. In univariate analysis, the median overall survival (OS) was associated with histological differentiation, invasion depth, lymph node metastasis, TNM stage and the expression of Shh, Ptch1, Smo and Gli1 (P<0.05, Fig. 2). Furthermore, multivariate survival analysis demonstrated that lymph node metastasis, TNM stage and a positive expression of Gli1 were independent prognostic factors for OS (P<0.05). These analyses are presented in Table 4. These results indicate that patients with the overactivation of the Shh pathway have a worse prognosis.

### Elevated expression of Gli1 in GC cell lines

**Figure 3:**
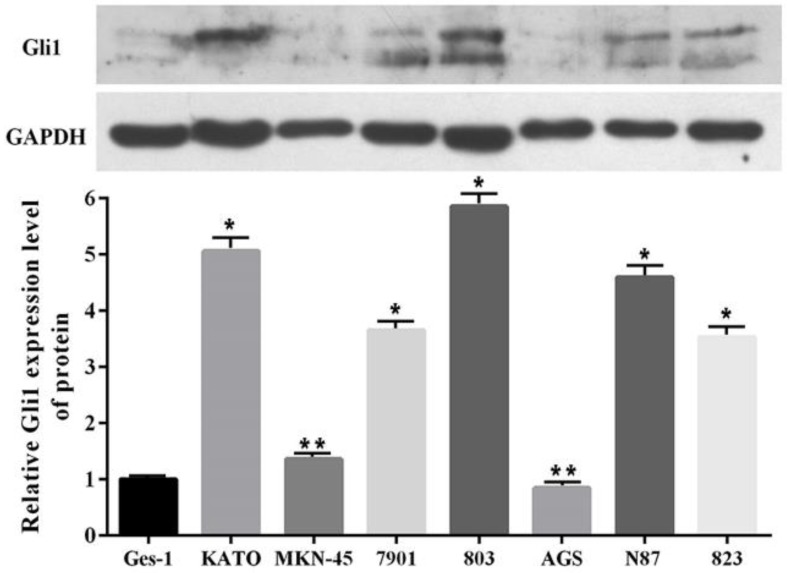
Gli1 protein expression level evaluated by western blot analysis in GC cells and GES-1 cells. GAPDH was used as an internal control. ^*^P<0.05, ^**^P≥0.05, compared with GES-1. GC, gastric cancer.

In order to more deeply understand the biological function of the Shh pathway in GC, the expression levels of Gli1 protein in seven GC cell lines and one normal human gastric epithelial cell line were examined. Western blot analysis revealed that the Gli1 protein expression level was higher in six of the GC cell lines (KATO, MKN-45, SGC-7901, MGC-803, N87 and BGC-823) than in the GES-1 cell line, but not in the AGS cell line (Fig. 3). In particular, its expression was most markedly upregulated in the MGC-803 cell line. Therefore, the MGC-803 and AGS cell lines were selected for use in subsequent assays.

### Shh/Gli1 pathway promotes GC cell migration and invasion

**Figure 4:**
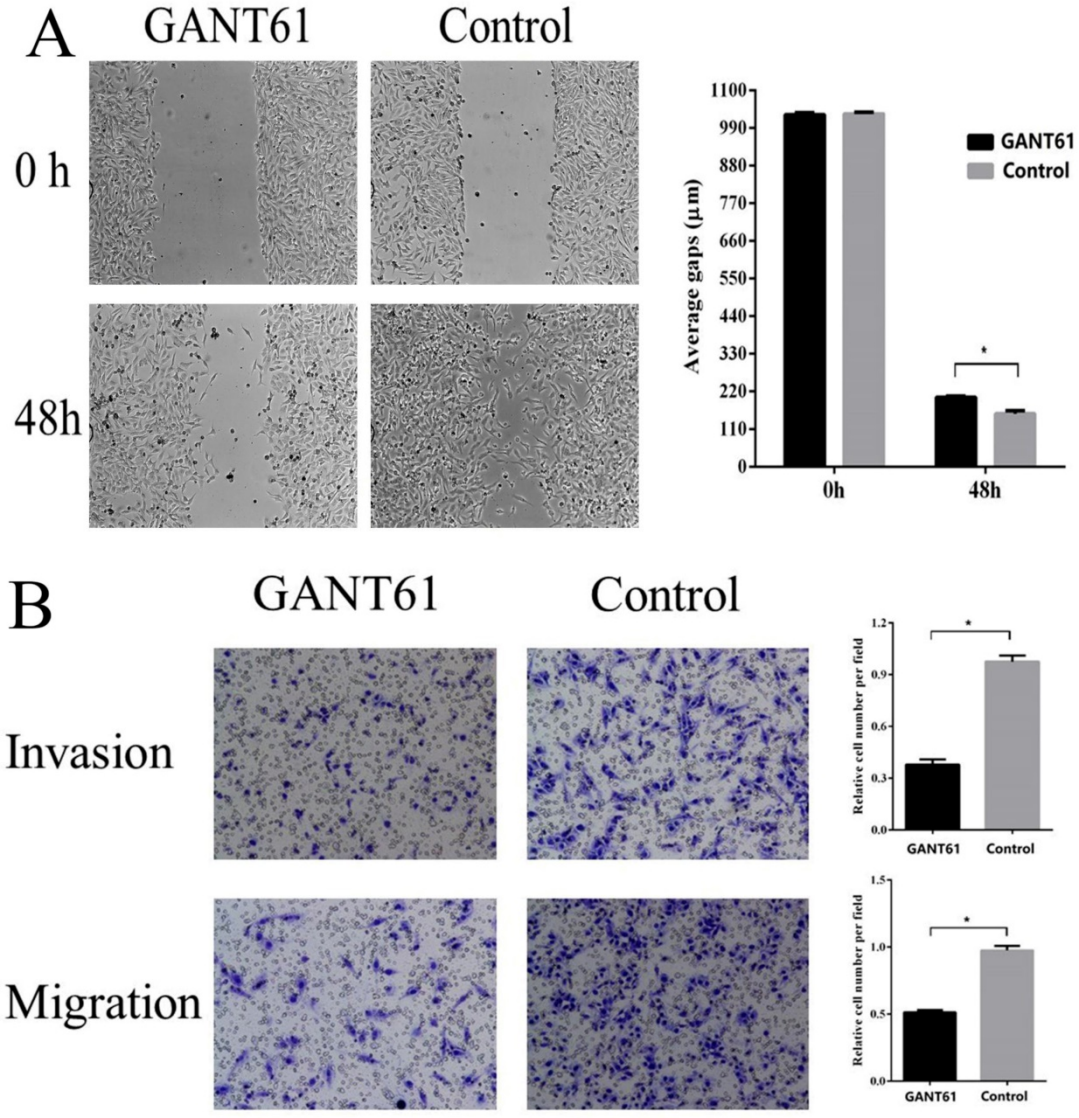
GANT61 inhibits MGC-803 cell migration and invasion *in vitro*. (A) Wound healing assay was performed to investigate the effect of GANT61 interference on MGC-803 cell migration. (B) Transwell migration and Transwell invasion assay were carried out to detect the migratory and invasive abilities of the MGC-803 cells treated with GANT61. MGC-803 cells were incubated with DMSO as a control. ^*^P<0.05.

**Figure 5:**
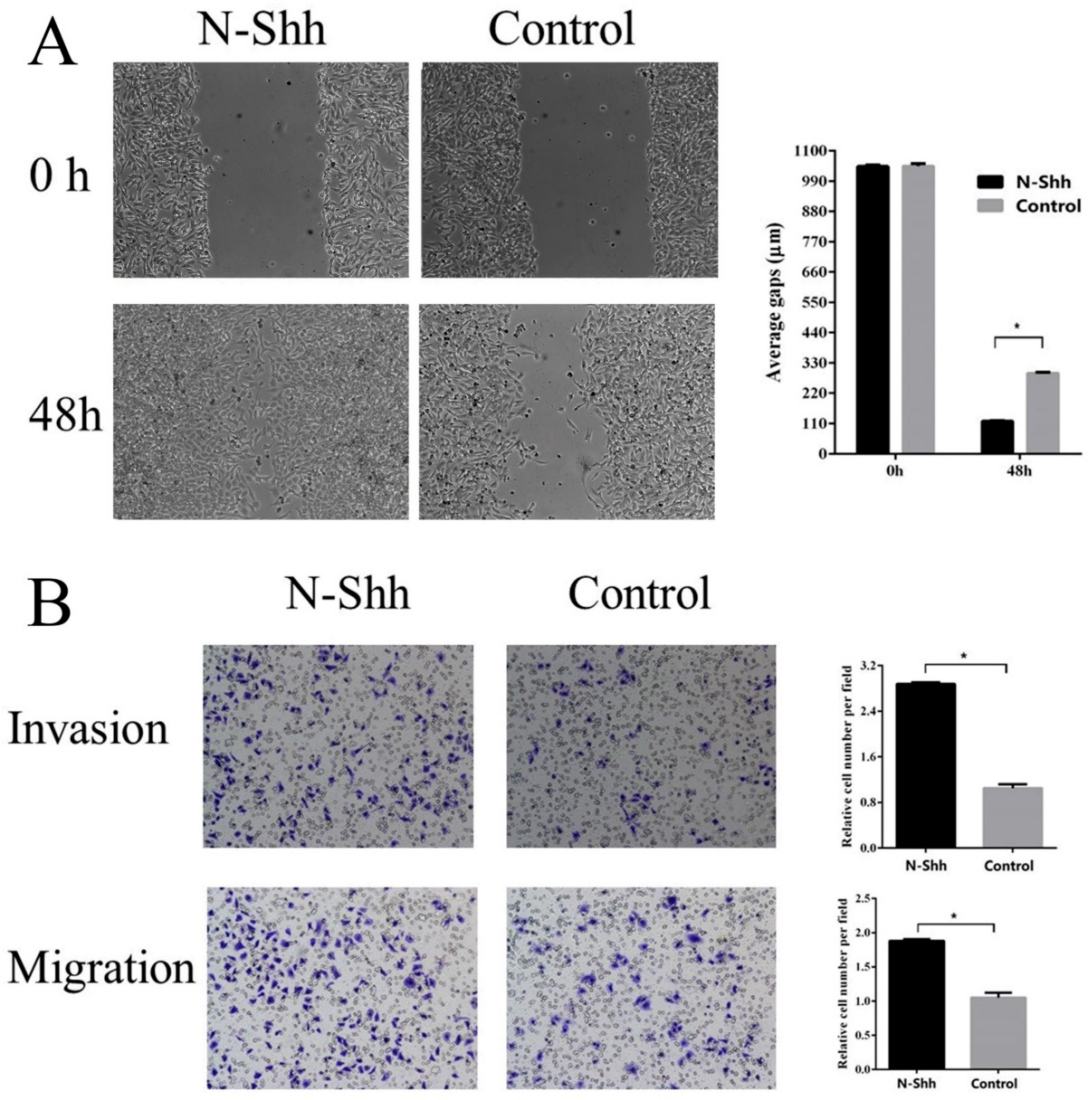
N-Shh promotes AGS cell migration and invasion *in vitro*. (A) Wound healing assay was performed to investigate the effect of N-Shh interference on AGS cell migration. (B) Transwell migration and Transwell invasion assay were carried out to detect the migratory and invasive abilities of AGS cells treated with N-Shh. AGS cells were incubated with DMSO as a control. ^*^P<0.05.

To examine the possible roles of the Shh/Gli1 pathway in regulating cell migration and invasion, wound-healing and Transwell assays were performed. GANT61 was used to block the Shh/Gli1 pathway in MGC-803 cells, and used N-Shh to stimulate the Shh/Gli1 pathway in AGS cells. MGC-803 cells treated with GANT61 exhibited a significant inhibition of cell migration in the wound healing assay and Transwell migration assay (Fig. 4); similarly, the invasive ability of the MGC-803 cells was significantly inhibited following treatment with GANT61 in the Transwell invasion assay. On the other hand, wound-healing assay and Transwell assay indicated that the AGS cells stimulated with N-Shh exhibited a significantly enhanced migratory and invasive capacity (Fig. 5).

### Gli1 promotes EMT in GC

**Figure 6:**
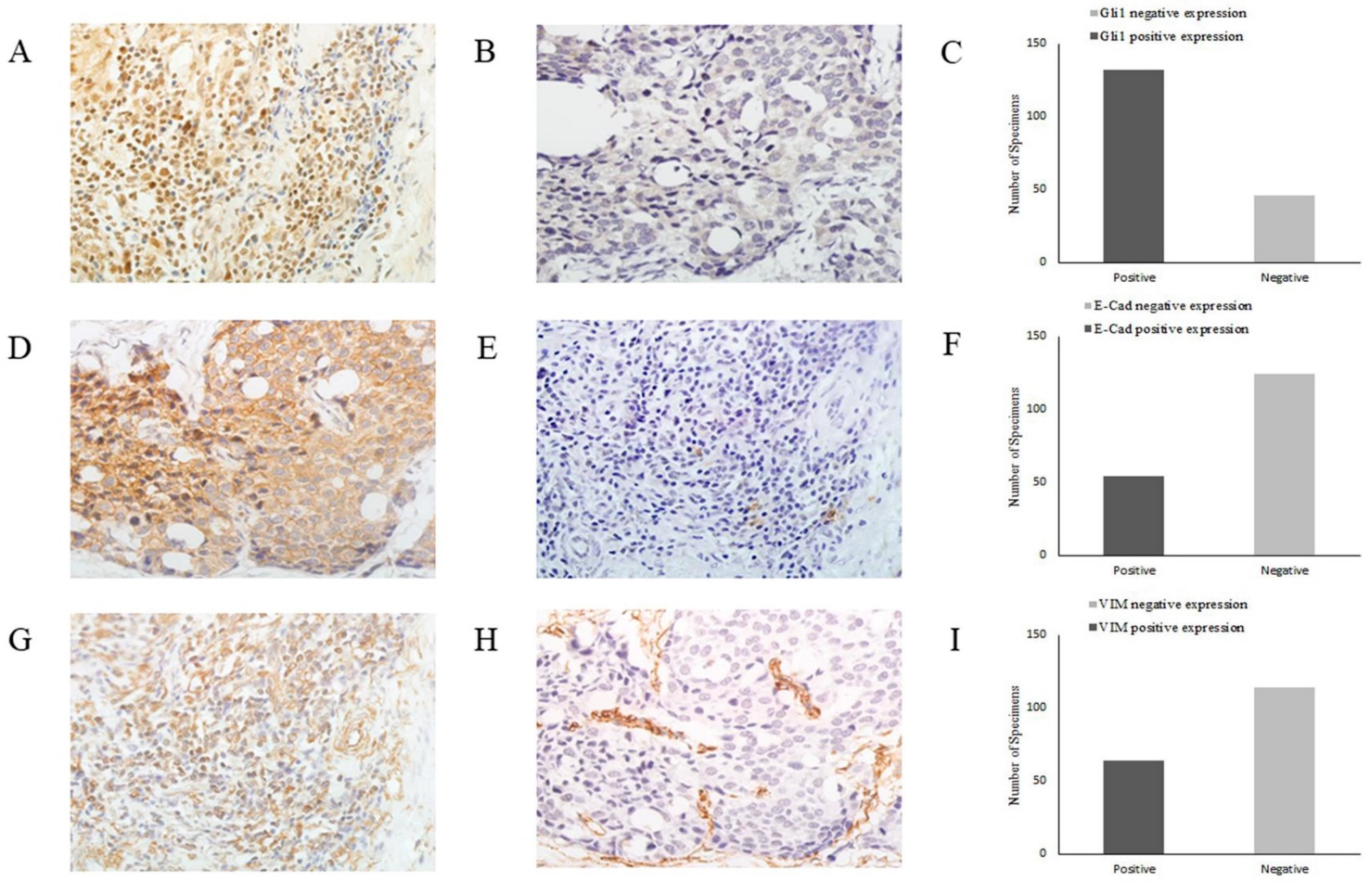
Representative images of Gli1, E-cad and VIM expression in GC tissues by immunohistochemistry (magnification, x400). (A) Gli1 positive expression in GC tissues, (B) Gli1 negative expression in GC tissues, (C) Relative Gli1 expression in GC tissues, (D) E-Cad positive expression in GC tissues, (E) E-Cad negative expression in GC tissues, (F) Relative E-Cad expression in GC tissues, (G) VIM positive expression in GC tissues, (H) VIM negative expression in GC tissues, (I) Relative VIM expression in GC tissues. GC, gastric cancer.

As EMT is crucial for cancer cell migration and invasion, in order to elucidate the role of the Shh/Gli1 pathway in the regulation of EMT in GC, the association between E-cad, VIM and Gli1 expression was investigated by immunohistochemistry in GC tissues (Fig. 6). The results are presented in Table 5. The association analysis revealed that the expression of Gli1 was significantly inversely associated with E-cad expression (OR=0.037; P=0.001) in GC tissues. By contrast, the expression of Gli1 and VIM exhibited a marked positive association (OR=2.937; P<0.01).

**Figure 7:**
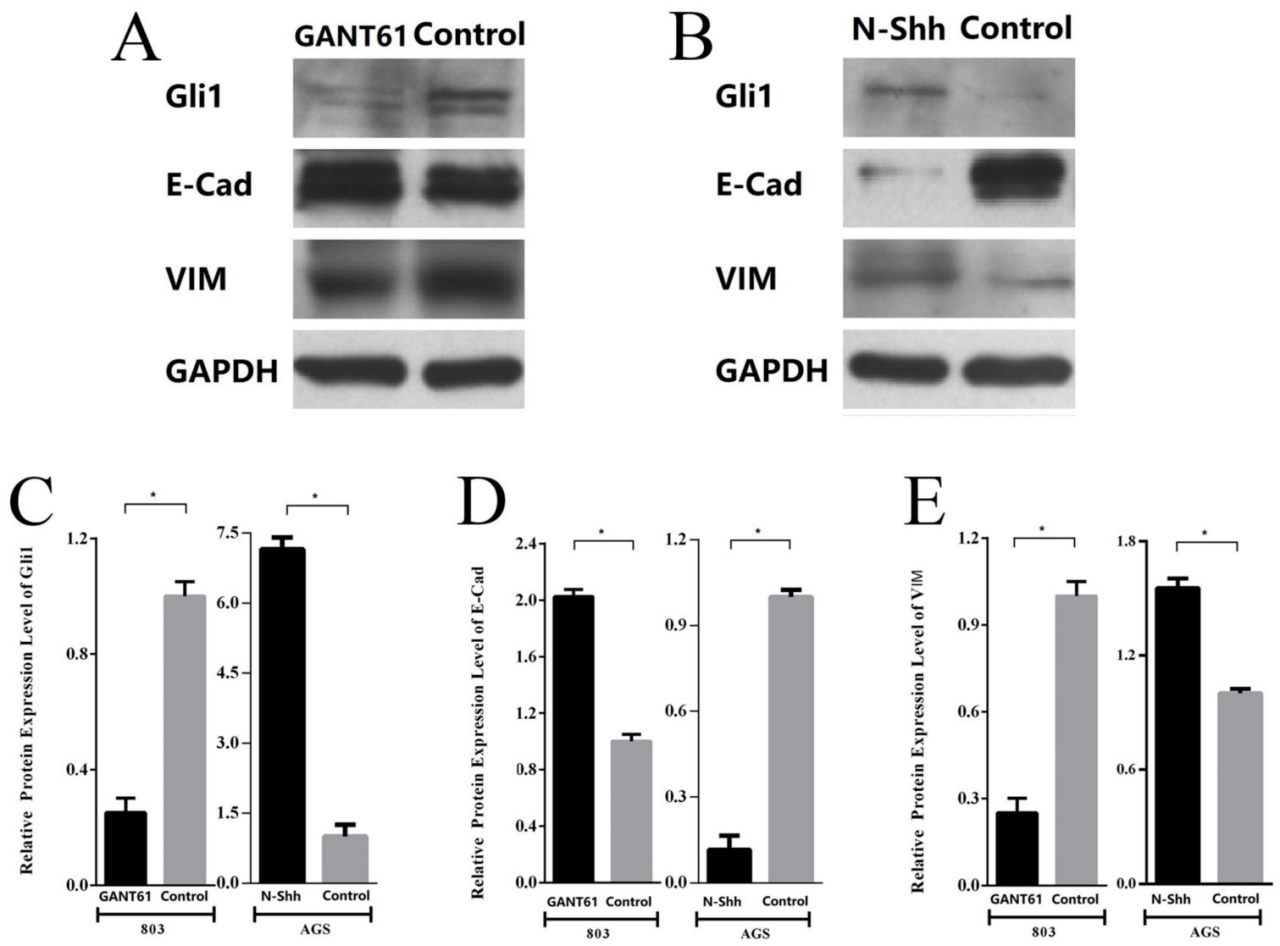
Shh/Gli1 pathway affects the expression of EMT markers in GC cells. (A) Western blot analysis was used to investigate the protein expression levels of Gli1, E-cad and VIM in MGC-803 cells treated with GANT61; (B) western blot analysis was used to investigate the protein expression levels of Gli1, E-cad and VIM in AGS cells treated with N-Shh; (C) relative protein expression level of Gli1; (D) relative protein expression level of E-Cad; (E) relative protein expression level of VIM. All values were normalized to GAPDH. ^*^P<0.05.

Subsequently, the changes in the expression of E-cad and VIM following the blocking or activation of Shh/Gli1 pathway activity in GC cells were detected. The results demonstrated that Gli1 expression was markedly downregulated in MGC-803 cells treated with GANT61. Western blot analysis also indicated that the E-cad levels were significantly upregulated with GANT61 treatment; however, the levels of VIM were downregulated (Fig. 7A; P<0.05). By contrast, following incubation with N-Shh, western blot analysis revealed that Gli1 and VIM expression levels were markedly elevated in AGS cells; however, the expression of E-Cad was decreased (Fig. 7B; P<0.05). These observations in GC cells were similar with the findings in GC tissues. These findings demonstrated that the aberrant activation of the Shh/Gli1 pathway may promote the EMT process, which may lead to the malignant behavior of GC cells.

## Discussion

The findings of this study illustrated that the Shh/Gli1 pathway was aberrantly activated in GC cell lines and tumor tissues, and that it contributed to the migration and invasion of GC cells. This study also indicated that there were a strong association between Gli1 and EMT markers in GC, suggesting that the Shh/Gli1 pathway may regulate GC progression and metastasis via EMT.

GC is among the most prevalent malignant tumors worldwide, with a relatively high morbidity and mortality rate. Tumor invasion and metastasis are the major reasons for the mortality of patients with GC 4. Therefore, it is imperative to explore novel diagnostic markers and therapeutic targets with which to promote early diagnosis and improve survival. An increasing number of studies have indicated that the Shh pathway is aberrantly activated in several types of cancer and plays a critical role in cancer growth, invasion and metastasis 29-31. Recent studies have demonstrated that the Shh pathway is overactivated in GC, and that it is associated with an aggressive biological behavior 13-15. However, the association between Shh pathway components and the clinicopathological features and prognosis of patients with GC remains contentious. Chen *et al* 15 demonstrated that Shh and Gli1 were overexpressed in human GC tissues and were associated with deeper cancer invasion, an increased likelihood of lymph node metastasis, and a poor pTNM stage. Lu *et al* 32 also reported similar results. By contrast, Saze *et al* 33 reported that these key Shh pathway members were significantly overexpressed in GC tissues, although there were no significant association between these members and the various clinicopathological parameters in GC. In addition, only the overexpression of Ptch1 was associated with a poor prognosis. It should be mentioned that the majority of these studies did not distinguish whether patients underwent radical resection or palliative surgery. In order to exactly confirm the association between Shh pathway components and GC clinical characteristics and outcome, all specimens analyzed in this study were from patients who had undergone radical resection. The results indicated that expression of Shh, Ptch1, Smo and Gli1 protein was upregulated in GC tissues in comparison with adjacent non-tumor tissues. There was a positive association between the upregulation of these markers and a deeper invasion depth, positive lymph nodal metastasis and a more advanced tumor stage. The overexpression of these markers was closely associated with an unfavorable prognosis of patients with GC. The Gli1 status was an independent prognostic indicator for survival of patients with GC. Additionally, the association analysis revealed that there was a significant positive association between every two members. These findings suggest that the Shh pathway is aberrantly activated and contributes to cancer progression and metastasis in GC. Gli1 can thus be used as a prognostic indicator for GC patients following radical resection.

It is widely considered that Gli1 is an important transcription factor in the canonical Shh pathway, which regulates the expression of a variety of downstream target genes 34. Thus, Gli1 is considered to be a symbol of Shh pathway activation. Consistent with the tissue expression analysis, the results of this study revealed that the Gli1 level in GC cell lines was evidently higher than that in the GES-1 cell line. To better explore the function of the Shh/Gli1 pathway in the regulation of GC cell behavior, the Shh/Gli1 pathway was blocked or activated in GC cells to investigate its functional effects on cell migration and invasion. GANT61 is a specific antagonist of the Shh/Gli1 pathway, which has an inhibitory effect on the transcriptional activity of GLI proteins 35. Previous studies have demonstrated that GANT61 suppresses the ability of cell migration and invasion, which was identified in hepatocellular carcinoma, pancreatic carcinoma and prostate cancer, as well as GC 29, 36-39. Consistently, the present study revealed that GANT61 can effectively inhibit Shh pathway activation in MGC-803 cells. Gli1 expression was markedly decreased following treatment with GANT61, and the cell migratory and invasive abilities were was also significantly suppressed. By contrast, N-Shh was used to activate the Shh/Gli1 pathway. When the AGS cells were stimulated with N-Shh, Gli1 expression was significantly increased, and N-shh markedly enhanced the migratory and invasive abilities of the AGS cells. Overall, these data strongly suggest that the Shh/Gli1 pathway has an essential function in regulating the migration and invasion of GC cells.

EMT is an intricate programme through which epithelial cells are transdifferentiated into mesenchymal-like cells 17, 18. An important characteristic of EMT is the loss of the expression of epithelial surface markers, mainly E-Cad, and a gain in the expression of mesenchymal markers, such as VIM 19, 20. Previous studies have demonstrated that EMT usually leads to pathological migration and invasion in the course of GC progression 40, 41. Although the exact mechanism of EMT is still under investigation, certain studies have suggested that a number of pathways may contribute to the regulation of EMT. Li* et al* 42 found that Gli-1 can upregulate the expression of the transcription factor Snail, with a consequent decrease in E-cad expression. Yoo *et al* 13 found that the Shh pathway promoted EMT progression through the upregulation of the expression of various EMT regulators via the TGF-ß pathway. These findings indicate that the Shh pathway may have a stronger association with EMT. In the present study, the results demonstrated that Gli1 expression was positively associated with VIM expression, and negatively associated with E-cad expression in GC tissues. Furthermore, this study revealed that E-Cad expression was upregulated and VIM expression was downregulated in MGC-803 cells when the Shh/Gli1 pathway was blocked by GANT61. Conversely, after the AGS cells were stimulated by N-Shh proteins, E-Cad expression was decreased, whereas the expression of VIM was significantly increased. Therefore, the above-mentioned results indicate that Shh/Gli1 pathway activation can induce EMT, which leads to GC progression and metastasis.

In conclusion, the current study illustrated that the Shh/Gli1 pathway is aberrantly activated in GC, and the overactivation of the Shh/Gli1 pathway is associated with a higher aggressive tumor phenotype and a poorer prognosis of patients with GC. Furthermore, the results indicated that the Shh/Gli1 pathway can enhance cell migratory and invasive abilities *in vitro,* possibly by inducing EMT. These results indicate that the Shh/Gli1 pathway may play a critical role in the development and progression of GC and may thus be regarded as a novel therapeutic target for GC.

## Figures and Tables

**Table 1:** Expression of Shh, Ptch1, Smo and Gli1 in GC tissues and adjacent non-tumor tissues.

Group	GC tissue (%)	Adjacent normal tissue (%)	χ^2^	P-value
Shh			19.985	0.000
Positive	128(71.9%)	39(43.8%)		
Negative	50(28.1%)	50(56.2%)		
Ptch1			19.908	0.000
Positive	119(66.9%)	34(38.2%)		
Negative	59(33.1%)	55(61.8%)		
Smo			4.690	0.030
Positive	101(56.7%)	38(42.7%)		
Negative	77(43.3%)	51(57.3%)		
Gli1			36.544	0.000
Positive	132 (74.2%)	32(36.0%)		
Negative	46(25.8%)	57(64.0%)		

**Table 2:** Association between the expression of Shh pathway components in GC tissues.

	Ptch1		OR	95%CI	P value	Smo		OR	95%CI	P value	Gli1		OR	95%CI	P value
	Positive	Negative				Positive	Negative				Positive	Negative			
Shh			2.765	1.402-5.452	0.003			2.302	1.183-4.479	0.013			2.293	1.125-4.671	0.021
Positive	94	34				80	48				101	27			
Negative	25	25				21	29				31	19			
Ptch1								1.949	1.036-3.667	0.038			5.068	2.477-10.370	0.000
Positive						74	45				101	18			
Negative						27	32				31	28			
Smo													2.635	1.322-5.250	0.005
Positive											83	18			
Negative											49	28			

OR: odds ratio, CI: confidence interval

**Table 3:** Association between Shh, Ptch1, Smo and Gli1 expression and clinicopathological parameters of patients with GC.

Variables	Cases (n)	Shh		P value	Ptch1		P value	Smo		P value	Gli1		P value
		Positive	Negative		Positive	Negative		Positive	Negative		Positive	Negative	
Gender				0.723			0.390			0.222			0.976
Male	128	93	35		88	40		69	59		95	33	
Female	50	35	15		31	19		32	18		37	13	
Age (years)				0.609			0.188			0.866			0.588
<60	126	92	34		88	38		72	54		92	34	
≥60	52	36	16		31	21		29	23		40	12	
Tumor size (cm)				0.484			0.271			0.310			0.564
<5	71	49	22		47	24		37	34		51	20	
≥5	107	79	28		72	35		64	43		81	26	
Histological differentiation				**0.012**			**0.002**			0.513			**0.047**
Differentiated	60	36	24		31	29		32	28		39	21	
Undifferentiation	118	92	26		88	30		69	49		93	25	
Serosal invasion				**0.042**			**0.038**			0.620			**0.000**
Negative	33	19	14		17	16		20	13		16	17	
Positive	145	109	36		102	43		81	64		116	29	
Lymph node metastasis				**0.040**			**0.026**			**0.023**			**0.012**
Negative	45	27	18		24	21		20	25		27	18	
Positive	133	101	32		95	38		81	52		105	28	
TNM stage				**0.019**			**0.018**			**0.030**			**0.004**
Ⅰ+Ⅱ	52	31	21		28	24		23	29		31	21	
Ⅲ	126	97	29		91	35		78	48		101	25	

**Table 4:** Univariate and multivariate analyses of the OS of GC patients following radical resection.

Variables	Univariable analysis			Multivariable analysis	
	HR (95% CI)	*P* Value		HR (95% CI)	*P* Value
Gender	0.903	0.599			
(Male vs. Female)	(0.615-1.323)				
Age (years)	1.289	0.178			
(<60 vs. ≥60)	(0.891-1.865)				
Tumor size (cm)	0.713	0.079			
(<5 vs. ≥5)	(0.489-1.040)				
Histological differentiation	2.212	**0.001**		1.447	0.199
(Differentiated vs. Undifferentiation)	(1.382-3.538)			(0.910-2.301)	
Serosal invasion	3.206	**0.000**		0.844	0.705
(Negative vs. Positive)	(1.491-6.891)			(0.351-2.032)	
Lymph node metastasis	2.084	**0.003**		1.724	**0.026**
(Negative vs. Positive)	(1.466-2.962)			(1.068-2.782)	
TNM stage	3.806	**0.000**		2.958	**0.000**
(Ⅰ+Ⅱ vs. Ⅲ)	(2.420-5.984)			(1.680-5.206)	
Shh expression	1.729	**0.025**		0.825	0.326
(Negative vs. Positive)	(1.070-2.792)			(0.562-1.211)	
Ptch1 expression	1.980	**0.011**		1.295	0.216
(Negative vs. Positive)	(1.337-2.933)			(0.860-1.950)	
Smo expression	1.416	**0.039**		1.050	0.810
(Negative vs. Positive)	(0.986-2.032)			(0.706-1.560)	
Gli1 expression	2.207	**0.000**		1.565	**0.016**
(Negative vs. Positive)	(1.423-3.422)			(1.070-2.792)	

HR, hazard ratio; CI, confidence interval

**Table 5:** Association between Gli1 with E-cad and VIM in GC tissues.

Variable	Gli1		OR	95%CI	P-value
	Positive	Negative			
E-Cad			0.307	0.152-0.621	0.001
Positive	31	23			
Negative	101	23			
VIM			2.937	1.311-6.578	0.007
Positive	55	9			
Negative	77	37			

OR: odds ratio, CI: confidence interval

## Abbreviations

GC: gastric cancer
Hh: Hedgehog
Shh: Sonic hedgehog
Path: Patched receptor
SMO: Smoothened receptor
N-Shh: recombinant Shh proteins
GANT61: Gli-antagonist 61
E-Cad: E-cadherin
VIM: Vimentin
EMT: Epithelial-to-mesenchymal transition
